# State-of the art methodologies dictate new standards for phylogenetic analysis

**DOI:** 10.1186/1471-2148-13-161

**Published:** 2013-08-01

**Authors:** Maria Anisimova, David A Liberles, Hervé Philippe, Jim Provan, Tal Pupko, Arndt von Haeseler

**Affiliations:** 1 Department of Computer Science, Swiss Federal Institute of Technology (ETH), Zürich 8092, Switzerland; 2 Swiss Institute of Bioinformatics (SIB), Lausanne, Switzerland; 3 Department of Molecular Biology, University of Wyoming, Laramie, WY 82071, USA; 4 Departement de Biochimie, Université de Montréal, Montréal, Qc H3C 1J7, Canada; 5 School of Biological Sciences, Queen’s University Belfast, Belfast BT9 7BL, UK; 6 Department of Cell Research and Immunology, George S. Wise Faculty of Life Sciences, Tel Aviv University, 69978, Tel Aviv, Israel; 7 Center for Integrative Bioinformatics Vienna, Max F. Perutz Laboratories, University of Vienna, Medical University of Vienna, Dr.-Bohr-Gasse 9, A-1030, Vienna, Austria

## Abstract

The intention of this editorial is to steer researchers through methodological choices in molecular evolution, drawing on the combined expertise of the authors. Our aim is not to review the most advanced methods for a specific task. Rather, we define several general guidelines to help with methodology choices at different stages of a typical phylogenetic ‘pipeline’. We are not able to provide exhaustive citation of a literature that is vast and plentiful, but we point the reader to a set of classical textbooks that reflect the state-of-the-art. We do not wish to appear overly critical of outdated methodology but rather provide some practical guidance on the sort of issues which should be considered. We stress that a reported study should be well-motivated and evaluate a specific hypothesis or scientific question. However, a publishable study should not be merely a compilation of available sequences for a protein family of interest followed by some standard analyses, unless it specifically addresses a scientific hypothesis or question. The rapid pace at which sequence data accumulate quickly outdates such publications. Although clearly, discoveries stemming from data mining, reports of new tools and databases and review papers are also desirable.

## Background and motivation

Phylogenetic analyses of molecular sequences are an integral part of many modern molecular and evolutionary biology studies. With the increasing pace of methodological developments it becomes a challenge for those authors that merely apply statistical methods to make sufficiently educated choices of what models and methods are most suitable for their data and purposes. As editors, we regularly come across submissions in which outdated methods are used with no apparent reason, undermining the reliability of reported findings. For example, most of the time no justification is provided for the use of alignment methods, typically with default settings followed by subjective manual intervention. Other common issues include the use of overly simplistic substitution models or reliance on basic pairwise comparisons when multiple homologous sequences are available. In particular, with no justification, some authors rely solely on distance-based tree reconstruction and, worryingly, statistical support for inferred clades is not properly evaluated. Further downstream, selection or dating analyses are common, but again, they often suffer from the use of outdated methods that are based on pairwise comparisons or make overly simplistic assumptions.

While researchers in the field are somewhat critical of outdated methods, in fact, many of them made and still make a profound contribution to the development of methodologies for computational molecular evolution, which explains their frequent usage. However, the field has since moved on and now boasts an overwhelming variety of more advanced models and methods, which were shown to be either better (more accurate) than previous methods in general, or to deal better with data-specific features. Appropriate application of this existing variety, nevertheless, requires a better understanding of the fundamental principles of the various methods and models, their underlying assumptions, and how they are implemented in various programs and web-servers. Looking forward, methods and strategies that are currently the state-of-the-art are likely to become outdated as well, so it is equally important to think broadly about the analysis performed. The field of molecular evolution is extremely interdisciplinary, bridging mathematics and statistics, computer science, ecology, evolutionary biology and population genetics, molecular biology, biochemistry, and physical chemistry. Few researchers have expertise in all of these areas, yet an analysis in molecular evolution is ultimately interdisciplinary, making assumptions across several areas, which may be not fully comprehended by a researcher undertaking the analysis. We appreciate that often, model and method choice is not a trivial task even for method developers. As a consequence, there has been a lot of recent effort in evaluating methods and models

## The classical phylogenetic analysis pipeline

Phylogenetic analysis of a set of sequences typically commences with the identification of homologous sequences. Next, a multiple sequence alignment (MSA) is constructed. This is often followed by phylogeny inference, which usually requires a substitution model. Further analyses and inferences may use other more sophisticated methods and models, which then rely on the inferred phylogeny. For protein-coding genes, a typical task involves estimating selective pressure on the protein. Ideally, all these steps should be performed simultaneously, since, for instance, an MSA provides crucial information to detect homologs and can only be correctly evaluated in the light of phylogeny. Due to computational complexity and burden, software allowing joint analyses such as the simultaneous inference of alignment and phylogeny are rare and will not be discussed here, although they clearly constitute an important avenue of research.

## The need for evolutionary motivation

Assume that an evolutionary pipeline has been established, and all methodological aspects have been appropriately considered: does this merit publication? If these are the *only* results reported in the manuscript then the answer is clearly no. In order for the analysis to be meaningful, the authors must clearly demonstrate that novel insights into the taxonomy or biology of studied organisms or the biology or biochemistry of specific molecular sequences were gained as a result. They should explain the choice of molecular data, list open questions that motivated the study, and define the hypotheses to be tested. For example, simply stating that an analyzed enzyme plays a central role in a given pathway is an insufficient justification. Likewise, plain inference of species relationships may be of little interest if the resulting tree does not help explain evolutionary processes along this tree or has no clear practical applications.

## The need for method justifications

One basic requirement for any phylogenetic analysis is to provide *justification* of the methodological choices taken, from a biological, biochemical, and/or statistical perspective. In general, a method should be selected because it was shown to be either superior or as good as its alternatives, with relevant studies cited. Another strong argument for method choice includes the ability of the chosen method to reflect the features of the data being analysed, and to address specific biological questions. Below we provide more specific advice regarding decisions to be made on the different stages of phylogenetic analyses.

## The benefit of using alternative methodologies

It has often been shown that phylogenetic conclusions might reflect bias in the methodology used. Although extensive research has detected and characterized biases in phylogenetic methods, there are likely to be many unknown biases, which may vary among methods. For example, one specific method or model can lead to biased results when too few taxa are analyzed, while another may be less accurate when sequences with high GC content prevail. Moreover, many methods are uninformative for sequence homologs when their divergence is too low or too high. It is thus the responsibility of the researchers to show that their conclusion is general rather than reflecting the outcome of one possible methodology out of equally good alternatives. It should be emphasized that the need for using alternative methodologies cannot be used as a justification to use outdated methodologies. Instead, only when two or more well-performing methodologies exist, there is a benefit to evaluate the dependence of the results on the choice of alternative methodologies. We demonstrate this benefit in the case of MSA algorithms, below. It is reassuring when a result consistently holds for several relevant methods or models. However, if the result is sensitive to the choice of models that fit the data similarly or methods that are known to be similarly accurate, caution should be taken when interpreting the results or when the results are used for downstream analyses. Ideally, one should aim to understand the underlying assumptions of the methodologies and discuss why they led to contrasting results.

## Reliability and data filtering

In a standard phylogenetic pipeline, the outcome of statistical inference at one step serves as input to the analysis at the next step. However, a single outcome (for example the ‘best’ MSA) is only a tiny fraction of the population of possible outcomes (the set of all possible MSAs). Pipelines should ideally be replaced by a probabilistic joint analysis of all relevant parameters. However, as this is almost never possible, pragmatic inference from pipelines should be conducted by methodologies that account for all uncertainties in all stages. For example, when inferring positive selection, it would be more accurate to base inferences on all possible/plausible alignments, models, model parameters, and trees than to base them on one or a subset of possible alignments. While accounting for all possible scenarios is seldom feasible, many recent methodologies allow accounting for uncertainty when analyzing data.

Bayesian methods allow integrating over uncertainty, for instance in phylogenetic inference. As a case in point, Bayesian tree search algorithms often integrate over the parameters of the assumed underlying model. When using Bayesian approaches, convergence diagnostics should be monitored and the influence of priors should be considered. While Bayesian approaches, when they exist, should clearly be considered, they may be extremely time consuming and so impractical for large datasets. But even considering just a few main (most probable) competing outcomes, may help to validate the robustness of final conclusions.

One computationally inexpensive strategy for dealing with the limitations of existing methods (for example to handle uncertainty) is to discard the data that are the most poorly explained by the models/methods used or that do not enable evaluation of the hypothesis or question being addressed. Thus, instead of averaging over possible MSAs, one can filter out unreliable alignment regions (that is, remove regions for which the methods used yield results with great uncertainty). Indeed, for some types of analyses, filtering out uncertainty was shown to be critical for accurate inference. For example, positive selection inference was found to be more accurate when unreliable MSA regions were filtered out. However, the use of filtering remains controversial; sometimes it can have detrimental impact on the accuracy of phylogeny inference, or introduce a systematic bias to the results by, for example, removing fast-evolving sites. On the other hand, filtering is sometimes justified by avoiding the perception of long branches attraction in systematic analysis. Thus, the use of filtering and the choice of appropriate filtering strategies should be carefully considered and justified by including relevant citations, as there are conflicting perspectives on this in the scientific community.

To summarize, researchers should make an effort to demonstrate their results are reliable and do not represent a tiny fraction of all possible evolutionary scenarios that could have led to the generation of their analyzed data. If the results vary depending on the methodology used, this should be reported rather than ignored as it allows *evaluation the uncertainty* of the inference and may help understand how methodological choices affect the resulting inference and conclusions.

## Reproducibility, data and code sharing and reporting

For science to progress and build upon previous work, the reader of a paper should be able to evaluate and repeat the analyses reported in the manuscript. Evolutionary studies are read by audiences with diverse sets of training. While standards in different fields may differ, authors should attempt to meet the standards of the different communities. To this end the methods should be detailed and ideally include, for example, the set of parameters and options used by the programs in the pipeline. If scripts or computer code were generated as part of the study, they should also be made available. Similarly, MSAs, trees, homologs and any other type of data included in an analysis should all be provided and ideally deposited in the relevant repository to facilitate reuse (see http://www.biomedcentral.com/about/editorialpolicies#DataandMaterialRelease). In this way, intermediate results within a pipeline can be made readily available so that, if a researcher comes up with a better way to perform one step in the pipeline, there is no need to repeat the entire pipeline afresh. A shift in thinking is needed so that useful data are not simply buried away in supplemental information or in additional files but curated in a suitable repository such as TreeBASE (http://treebase.org/) or, where an appropriate data-specific repository is not available, a generic repository such as Dryad (http://datadryad.org/). In this way data are discoverable, identifiable (with DOI) formatted for easy reuse, and updatable. (Researchers dealing with large-scale data may want to consider the ‘Semantic Web’, which is becoming a new standard for representation of biological data and knowledge.

## Common pipeline steps

### Stage 1. Detecting homologs

Often, finding a set of homologous sequences is the first step in an evolutionary analysis. The first important point to consider is the goal of the search. When the goal is to reconstruct a species tree, for most methods, only orthologs may be sought, because mis-identification of paralogs as orthologs can yield an incorrect result; however, a few methods reconstruct the species tree from the reconciliation of gene trees, overcoming the limitation of using orthologous sequences, and are therefore promising. Such methods are dependent upon the model and assumptions made during the reconciliation process. For studies of gene families, orthologs, paralogs and xenologs are needed. Another point to consider is whether to search for all homologous sequences or to limit the search to a specific group (for example only vertebrates or only mammals). Where the search is indeed limited to a specific group, it is necessary to explain the motivation behind such a decision. Finally, the outgroup sequences used to root the tree, when possible, should be carefully chosen. Notably, numerous publications about taxon and sequence sampling exist and considering this accumulated knowledge in these fields can help guide the search for homologous sequences.

Once the scope of the search is determined, there is the question of choosing the query sequences for the homologous sequence search. When searching for all homologs in a specific gene family, a single BLAST search of the human sequence against a standard database may miss many homologs. It is likely that another search, starting from sequences identified in the first run would lead to the detection of additional homologs. In this respect, homology detection is often an iterative procedure in which sequences identified at each step are used to refine the search. The search stops when no new homologs are detected. Taking into account context-dependency (non-independence of sites) can further increase the power of a homology search, identifying remote homologs.

While BLAST is clearly the most commonly used algorithm for homology search, there are many alternative methods that can potentially detect homologs missed and exclude unrelated homologs erroneously included by BLAST. These methods can be divided into sequence-based and sequence-structure-based methods. Within the sequence-based methods, we mention the psi-BLAST algorithm, other profile search algorithms, methods using a Hidden Markov Model, and other advanced machine-learning techniques. Within each method, one should remember that the default cost matrix used by the algorithm and the gap penalties may not be ideal for the specific data analyzed.

While structural information may aid the detection of remote homologs, a structure-based search should be carefully considered: structural similarity alone may identify sequences that are the result of convergent evolution, rather than sequences that evolved from a common ancestral molecule.

Finally, the end result of the search should be evaluated with regard to the research question at hand: is there enough data (for example have enough taxa been sampled) to answer the set of hypotheses? Should some sequences be filtered out in order to increase the reliability of the alignment?

### Stage 2. Multiple Sequence Alignment

The choice of an alignment method is critical to downstream analyses and should be considered carefully. Each alignment column is a statement of homology, representing the descent from a common ancestry. Several recent reviews have offered a detailed perspective on the field of alignment. Here we outline only some of the key issues in a phylogenetic pipeline.

The first consideration is the data that one will align. Alignment is most commonly performed at the DNA or the amino acid level. However, for protein-coding genes codon alignment is often necessary, for example for tasks which involve characterizing selection on the protein, or other codon-based analyses. In these cases, DNA alignment lacks codon structural information and it is typically preferred to align at the protein level and back translate amino acid gaps to three nucleotide gaps in the corresponding DNA sequences, resulting in a codon alignment. This assumes that frame shifts never happen, and statistical alignment approaches using codon models may be more robust to this assumption. Alignment with empirical codon matrices is now possible in a few software packages.

Structural alignment (sequence alignment that is guided by one or more available structures of the proteins or RNA being analyzed) is sometimes employed for more distantly related sequences. However, sequences can slide through structures during evolution and fit of a sequence to a structure assayed with a force field is not necessarily a statement of evolutionary history. This has the potential to lead to incorrect evolutionary inference if subsequent steps like tree building are performed at the sequence level using the alignment.

Different sequence-based alignment methods can also give very different results, due to differences in assumptions and statistical and algorithmic approaches. Further, the substitution matrix and gap penalties used for alignment scoring should be tuned to the divergence of the sequences being aligned. Once an alignment is obtained, software to identify sequences or regions that are poorly aligned can be applied. However, one should keep in mind that substitutions, insertions, and deletions happen in evolution and an alignment that does not minimize such events may still be evolutionarily correct. Caution should be taken with repetitive sequences, which may introduce highly variable regions within the alignment. Furthermore, repetitive sequences and mobile elements may be homoplasious, and may thus lead to false inference of homology.

For these reasons, alignments should not be manually adjusted, as this is subjective and therefore not repeatable. It can be justified to adjust alignments based upon expected conserved biochemical or structural features (with the explicit assumption that these are conserved consistent with the evolutionary homology and therefore can be used as alignment ‘anchors’). If this is done, the criterion and justification for doing so should be explicitly stated and the pre- and post- adjustment alignments should be included in supplementary materials.

### Stage 3. Quality control

The accuracy of a phylogenetic analysis does not depend only on the models and methods used, but also on the quality of the data. Multiple errors, such as taxon misidentification, sequencing error, annotation error or sequence contamination, can occur during data collection and lead to errors in public databases. Such errors are indeed becoming more frequent, because the level and efficiency of quality controls, which were often manual in phylogenetics, did not follow the flood of high-throughput data production and because publication pressure favors the release of draft, instead of complete, genomes.

The incorrect assignments of sequences to species are particularly problematic, because erroneous, yet strong, signals are included in the data matrix, potentially yielding deeply flawed results. These errors are due to an initial incorrect taxonomic identification or, more frequently, to a contamination. Contaminations can occur at the level of the biological sample (such as DNA from parasites), of the sequencing center (such as DNA from previously sequenced organisms) and of the computational processing. For instance, the very small contigs of a draft eukaryotic genome may in fact be of prokaryotic origin. It is therefore crucial to verify the correct taxonomic assignment of each sequence, especially in multi-gene phylogenetic inference, using phylogenetic congruence, nucleotide composition, codon usage and, if necessary, additional wet experiments.

Sequencing, assembly and annotation errors are also quite frequent. They are particularly detrimental to molecular evolution studies, since they can seriously inflate the number of sites inferred to evolve under positive selection or the number of insertion and deletion events. For instance, a frameshift or an incorrect exon prediction will create a long string of amino acids without similarity to those of other species, creating multiple indels and non-synonymous substitutions, even for a highly conserved region. Researchers should be aware of such potential errors, and whenever possible, aim to detect them. For example, two protein coding sequences from two diverged mammals which are identical both at synonymous and non-synonymous sites may indicate contamination.

Finally, even in the absence of errors, the accuracy of a phylogenetic inference is sensitive to the completeness of the alignment. As the effective number of taxa (hence the ability to detect multiple substitutions) is directly related to the number of known states per site, the higher the amount of missing data, the higher the risk of tree reconstruction artifact. The existence of missing data is unavoidable, especially in a large data matrix, because of gene loss and difficulty in obtaining the sequences, but information about the amount and distribution of incompleteness should be clearly stated and their potentially misleading effects should be studied and/or carefully discussed.

### Stage 4. Model selection

It is important to remember that all evolutionary models are approximations of the course of evolution and thus a model can never be considered as ‘truth’. There is always a balance between over-simplified models and models which over-fit the data. Over-simplified models often ignore important aspects of the data and may lead to biased conclusions. In contrast, models that use too many parameters may over-fit the specific data, which can result in large errors in estimated parameters. In addition, over-fitting models may capture patterns that are specific to the data analyzed, and may thus lead to conclusions that do not reflect the population from which the data were sampled. Thus, the number of parameters should be tuned based on the dataset size, with larger datasets (which are becoming more frequent) allowing for parameter-rich models. Choosing the ‘right’ model for specific data is not a trivial task and thus, model selection procedures were developed in order to find the best model.

To summarize, models used in phylogenetic analyses should be justified. Notably, often more than one model can fit the data equally well, because they will handle different evolutionary properties more adequately (for example codon structure or non-stationarity of nucleotide composition). Using several well-fitting models allows demonstration of the robustness of conclusions. This relates to our sequel point, which is the benefit of using multiple methods and models to analyze a specific dataset, especially if mechanistically-motivated models are not available. It should be noted that the most widely used software for model selection analyze only a limited diversity of models, for example variations of the GTR+I+Γ model. However, numerous alternative models that incorporate heterogeneity of the substitution process across sites and/or over time or codon structure are available and were generally shown to fit the data better. They should therefore also be considered.

The model choice should ensure that the assumptions and the features of the model enable the inferences relevant to the study goals, such as testing of specific biological hypotheses. Once a suitable model (or a set of models) has been selected, it is important to show that the model adequately describes the data under scrutiny. Indeed, goodness-of-fit tests are common practice in statistics and have been widely applied in phylogenetics. Notably, testing the adequacy of a model is not always straightforward. It is important to test the relationship between model parameters and the biological processes studied. In addition, robustness to violation of certain model assumptions may also be tested. Conclusions drawn from non-fitting models should be discussed with care. Alternatively, the researcher can apply recently developed tools to identify the misfitting parts in the data.

When relevant and possible, combining data from different sources should be considered. One has to consider whether a concatenation of molecular sequences is sensible or whether different segments should be separately analyzed. It is important to remember that different sequence segments may evolve with different evolutionary patterns as they are affected by different mutational and selective constraints. This could be reflected in a combined dataset by defining data partitions. Here model choice for different data partitions in the combined dataset will be crucial for further interpretation of results. One can consider using mixture models or asking if a network rather than a tree describes the data in this context. Networks are particularly suitable for intraspecific studies, where ancestral haplotypes/genotypes may still be extant. However, the shorter coalescent times associated with such studies means that the use of multiple loci should be considered, since incomplete lineage sorting increases the chance that the genealogy of any single locus might not be fully representative of that of the species or populations under study. In addition, several types of data may be considered for specific analyses. For example, when inferring trees from genomic data it is possible that partitions that refer to protein sequences are analyzed at the amino-acid level while other partitions that refer to non-coding sequences are analyzed at the nucleotide level. Thus, when applicable, various coding and partitioning of the data should be considered and justified.

### Stage 5. Phylogeny inference

A vast number of evolutionary studies build and test their hypotheses based on inferred phylogenies, which should reflect the evolutionary history of a set of homologous sequences. Consequently, phylogeny inference became one of most standard tasks in evolutionary pipelines. Until the early nineties, parsimony and distance-based tree-building methods were preferred. More recently, probabilistic model-based methods, namely the maximum likelihood (ML) and the Bayesian approaches have grown to prominence due to their statistical properties and inferential powers. Moreover, these approaches go beyond simple phylogeny inference, providing a convenient statistical framework for further model selection and biological hypothesis testing. While parsimony is sometimes justified as model-free, it has mathematical properties and is not assumption-free; therefore explicit models should be generated for many biological problems. Likewise, distance-based methods may be unreliable for highly diverged data, yet they are often model-based and have nice mathematical properties and thus they may enable very fast and relatively accurate estimation of relevant biological parameters. Distance-based methods for tree reconstruction, such as neighbor joining, are extremely fast, and can provide reasonable solutions for extremely large data sets, something that would be much more computationally challenging with ML or Bayesian methods, even with recent computational advances. Furthermore, a candidate tree obtained with a distance method can be taken as a starting tree for ML heuristic searches. With the Bayesian approach, as a general rule, care should be taken to study the convergence diagnostics and the sensitivity of the estimates to prior distributions.

Since the conclusions of an evolutionary study rely on an inferred tree, the statistical support for the inferred tree or particular nodes should be reported. Luckily, the current arsenal of methods for branch support includes not only the traditional bootstrap and jackknife, but also a number of alternative methods. In particular, both Bayesian and ML programs for tree inference offer a variety of support values that are estimated along with a tree, such as posterior probabilities of clades or supports based on approximate likelihood tests. Once again, both the inferred tree and the support values of a specific node may change depending on the model assumed during the analysis. Failure of a model to account for major biological forces shaping the evolution of a sequence may lead to various systematic biases, such as the well-known Long Branch Attraction (LBA) artifact. Moreover, it is now well-documented that gene trees often do not coincide with species trees. The species trees concept has recently been questioned, particularly in prokaryotes. Considering a distribution of gene trees or a distribution of candidate trees for one specific DNA region can bring real insights into evolutionary biology, setting new standards for phylogenetic studies.

Testing some evolutionary hypotheses (for example, testing the monophyly of a group of species) may require a rooted tree. The choice of a root is not always trivial. The most common rooting method is via an outgroup, which should be selected and justified carefully. If data present a signal of non-homogeneous and non-reversible evolution in time (such as drifting GC content through evolution), it might be possible to infer the position of the root using non-reversible models. This however, increases the complexity of the estimation problem and requires large samples with informative sequence divergence. Formal gene tree/species tree reconciliation can also be used to root a tree, when a reference species tree is available.

Further, a weak or conflicting tree signal may be indicative of the biological factors that perturb the tree-like ancestral relationships. Possibilities of such events have to be considered and may include lateral gene transfers, recombination, gene conversion, incomplete lineage sorting, gene duplication and gene loss, and sequence convergence. While a tree representation is convenient for computational purposes, methods that relax this assumption already exist, such as phylogenetic network reconstruction.

### Stage 6. Inference of evolutionary selective forces

Many fundamental questions in evolutionary biology involve estimating the type and intensity of natural selection from molecular sequences. As with other tasks, methodological choices are crucial for the accuracy and power of the inference. Particularly, both ML and Bayesian approaches have been shown to be well-suited for evaluating selective pressures. Considering the state-of-the-art methodology, pairwise sequence comparisons have little value for investigating selection on specific genes. Instead, MSA-based analyses coupled with explicit evolutionary models enable estimating a variety of intricate details about the evolutionary process.

Selection analyses typically require a careful formulation of biological hypotheses to be tested. These will dictate the choice of methods and models and the types of analyses that are most appropriate for a particular case study. For example, focusing on lineage-specific selection on a protein will require using branch-specific models, while searching for specific residues under positive selection in a 3D structure will necessitate models allowing for variable selection pressure among sites or sites and branches together. Once again, the size and divergence of the dataset defines the limits to the power of the analyses. For instance, detecting episodic selection at a handful of residues may require a particularly large number of sufficiently diverged sequences. Note that hypotheses should be formulated *a priori* (before “’ooking’ at data), and cannot be based on estimates from a related statistical analysis. In molecular biology, positive selection is ultimately used as a predictor of lineage-specific functional change, in which case additional analysis might be desirable. It is known that compensatory co-variation within proteins can account for elevated lineage-specific rates of amino acid substitution and dN/dS (possibly linked to changes in population genetic parameters). Still, lineage-specific rate variation also contains signals for lineage-specific functional change in proteins and is valid as an imperfect predictor of functional change. Additional lines of structural and functional evidence involving observed substitutions is ultimately necessary to validate predictions of lineage-specific functional change.

Reliability of estimates and tests should be reported. Additionally, the possibility of known artifacts and model assumptions should be addressed: how would the results be affected by uncertainty in the alignment and phylogeny and various data biases (for example recombination, selection on synonymous positions, saturation of substitutions, codon usage preference, heterogeneous GC)? When the same hypothesis for positive selection is tested multiple times, a suitable correction is often necessary.

### Stage 7. Interpretation and conclusions

Even when all the analyses are completed, it is premature to conclude that the study is complete. The interpretation of the results is as important as its motivation and design. The study has little value without understanding the significance of results and putting them into a wider biological context. Here the first pre-requisite is a thorough knowledge of the literature concerning the system of interest: mining for additional evidence from the literature, experiments, or support from complimentary data is essential at this stage. Valuable insights may sometimes come from connecting previously disparate reports, and fully using additional available information, such as the paleontological record, functional and structural annotations, expression levels, ethological data, phenotypic characteristics, and protein sequence-structure-function studies (for example, those involving mutagenesis experiments). Indeed, a multitude of factors at play need to be considered to truly understand the workings of complex biological systems.

For example, to gain a meaningful interpretation of a phylogeny, the integrity of signals from ecosystem, development, physiology, protein structure and function, gene linkage, and other biological sources need to be considered. Similarly, studies of selective pressures on a lineage of a particular gene family may benefit from further analysis of the positively selected sites in the context of functional data and/or protein structure, including additional computational experiments using the techniques of structural bioinformatics. This may help to understand how natural selection actually happens in a protein.

## Conclusions

Above we have summarized what we consider to be the fundamental research practices that are recommended for a phylogenetic study to be useful and ultimately publishable. Primarily, before commencing any phylogenetic analyses, we advise a careful consideration of the biological motives driving the study and the formulation of evolutionary hypotheses to be tested. Once the biological motivation is clear, a suitable set of phylogenetic analyses can be included in a pipeline, where all methodology choices should be rigorously addressed. This can be done based on a sample of the latest methodology reviews, and can be particularly successful through inter-disciplinary collaborations. The interpretation of results is not less important than the study motivation and design. Drawing on the wealth of currently available additional data sources, the results should be interpreted in a wider biological context, clearly outlining the contribution to the field of biology. Manuscripts that we will consider for peer-review in *BMC Evolutionary Biology* should describe new biologically relevant methodologies or clearly show a significant advance to understand a biological question of general interest to the journal’s readership. We would also expect supporting data to be deposited in an appropriate repository.

## Recommended Reading

Anisimova, M (ed): *Evolutionary Genomics: Statistical and Computational Methods.* (in 2 volumes) New York: Humana Press: 2012

This edited volume provides a comprehensive overview of many state-of-the-art statistical and computational methods applied to sequence analysis in genomics.

Cannarozzi, GM, Schneider, A (eds): *Codon Evolution: Mechanisms and Models*. Oxford: Oxford University Press: 2012

This edited volume focuses on the application of methods for sequence analysis that incorporate the genetic code into DNA sequence analysis.

Felsenstein, J: *Inferring Phylogenies*. Sunderland, MA: Sinauer Associates Inc: 2003

This classic text overviews the development of the field of phylogenetics, quantitatively describing methods that have been developed for phylogenetic analysis.

Hall, BG: *Phylogenetic Trees Made Easy: A How To Manual*. City: Sunderland, MA: Sinauer Associates Inc: 2011

This user’s guide to phylogenetic tree software guides the novice biologist through phylogenetic analysis.

Nei, M, Kumar, S: *Molecular Evolution and Phylogenetics*. Oxford: Oxford University Press: 2000

This classic text extends upon phylogenetic analysis to quantitatively describe methods for sequence analysis, including the detection of selection.

Lemey, P, Salemi, M, Vandamme, A-M (eds): *The Phylogenetic Handbook: A Practical Approach to Phylogenetic Analysis and Hypothesis Testing*. Cambridge: Cambridge University Press: 2009

This text integrates descriptions of research strategies with practical information on phylogenetic analysis.

Nielsen, R (Ed): *Statistical Methods in Molecular Evolution*. New York: Springer: 2005

This edited volume also describes the statistical methods available for sequence analysis from the perspective of individual chapter authors.

Otto, SP, Day, T: *A Biologist's Guide to Mathematical Modeling in Ecology and Evolution*. Princeton: Princeton University Press: 2007

This is an introduction to the process of constructing models for evolutionary processes, intended for a biologist new to modeling.

Page, RDM, and Holmes, EC: *Molecular Evolution: A Phylogenetic Approach*. Malden, MA: Wiley-Blackwell: 1998

This is a classic text on phylogenetic and systematic analysis that introduces the field to biologists in a largely non-quantitative manner.

Semple, C and Steel, M: *Phylogenetics*. Oxford: Oxford University Press: 2003

This book provides a more mathematical description of problems in phylogenetics.

Yang, Z: *Computational Molecular Evolution*. Oxford: Oxford University Press: 2006

This text widens the discussion of phylogenetic analysis to quantitatively describe methods for sequence analysis and the detection of selection, extending the discussion of other books to include the widely used methods by the author.

